# Characterization of Fibrinogen as a Key Modulator in Patients with Wilson’s Diseases with Functional Proteomic Tools

**DOI:** 10.3390/ijms20184528

**Published:** 2019-09-12

**Authors:** Pei-Wen Wang, Tung-Yi Lin, Yu-Chiang Hung, Wen-Neng Chang, Pei-Ming Yang, Mu-Hong Chen, Chau-Ting Yeh, Tai-Long Pan

**Affiliations:** 1 Department of Medical Research, China Medical University Hospital, China Medical University, Taichung 40447, Taiwan; pwwang5105@hotmail.com; 2 Department of Traditional Chinese Medicine, Chang Gung Memorial Hospital, Keelung 20401, Taiwan; tungyi30@cgmh.org.tw; 3 Department of Chinese Medicine, College of Medicine, Kaohsiung Chang Gung Memorial Hospital and Chang Gung University, Kaohsiung 83301, Taiwan; e120845@cgmh.org.tw; 4 Departments of Neurology, College of Medicine, Kaohsiung Chang Gung Memorial Hospital and Chang Gung University, Kaohsiung 83301, Taiwan; cwenneng@ms19.hinet.net; 5 TMU Research Center of Cancer Translational Medicine, Taipei Medical University, Taipei 11042, Taiwan; yangpm@tmu.edu.tw; 6 Graduate Institute of Cancer Biology and Drug Discovery, College of Medical Science and Technology, Taipei Medical University, Taipei 11042, Taiwan; 7 Department of Psychiatry, Taipei Veterans General Hospital, Taipei 11217, Taiwan; kremer7119@gmail.com; 8 Department of Psychiatry, College of Medicine, National Yang-Ming University, Taipei 11221, Taiwan; 9 Liver Research Center, Chang Gung Memorial Hospital, Taoyuan 33375, Taiwan; chautingy@gmail.com; 10 School of Traditional Chinese Medicine, Chang Gung University, Taoyuan 33302, Taiwan; 11 Chinese Herbal Medicine Research Team, Healthy Aging Research Center, Chang Gung University, Taoyuan 33302, Taiwan; 12 Research Center for Chinese Herbal Medicine and Research Center for Food and Cosmetic Safety, College of Human Ecology, Chang Gung University of Science and Technology, Taoyuan 33303, Taiwan

**Keywords:** Wilson’s disease, ceruloplasmin, oxidative stress, fibrinogen, network analysis

## Abstract

Wilson’s disease (WD) is an autosomal recessive disorder of copper metabolism caused by defects in the ATPase gene (ATP7B). The various clinical features result from the massive accumulation of copper in the liver, cornea and basal ganglia. Although WD can be effectively treated with proper medicine, this disease is difficult to clearly diagnose due to its indefinite symptoms. In the current study, we achieved a positive correlation between clinical symptoms and the enzymatic activity of ceruloplasmin in WD patients. Furthermore, proteome profiles of plasma as well as network analysis demonstrated that fibrinogen is a critical indicator which is significantly unregulated in WD subjects in comparison to healthy donors and closely linked to pathogenesis of WD. Here, we applied 2DE-immunoblots and immunohistochemistry to verify the protein level and localization in situ. The enhanced expression of fibrinogen in the plasma of WD subjects with respect to that of healthy controls and patients with distinct disorders was also confirmed by utilizing clinical samples. As expected, application of high dose of copper induced expression of fibrinogen, while knockdown of ceruloplasmin also resulted in upregulation of fibrinogen as well as elimination of superoxide dismutase (SOD), leading to increased oxidative stress in cells. In summary, the liver injury or oxidative stress induced by the progression of WD may account for the obvious increase of fibrinogen, which in turn triggers inflammatory responses and interferes coagulation cascades; this finding sheds light on the early detection and diagnosis of WD.

## 1. Introduction

Wilson’s disease (WD) is hereditable and affects approximately one in 30,000 individuals worldwide, with exceptions found in specific subgroups with consanguinity [1]. The biochemical defect that causes WD is a deficiency of gene, encoding a copper transporting p-type ATPase (ATP7B), located on chromosome 13. Over hundred mutations of ATP7B, including point mutations, deletions and frame-shift, have been reported in patients with WD. Most patients have two different mutations of the gene on each allele encoding the WD gene, called compound heterozygotes [2,3]. This protein product is similar to other ATP-dependent transporters for heavy metals, which is mainly expressed in the liver and mediates copper homeostasis in liver cells.

Copper is an essential trace metal for living systems, however, cellular copper in excess of the needed quantity leads to oxidative stress and toxic effects [4,5]. Patients with WD are characterized by excessive accumulation of hepatic copper and a significant decrease in the plasma concentration of ceruloplasmin, thus suggesting reduced biliary excretion of copper and disturbed incorporation of copper during the biosynthesis of ceruloplasmin (CP) [5,6]. Affected patients usually present liver diseases such as inflammation, cirrhosis or fulminant liver failure. Neuropsychiatric symptoms are common in adults with WD, including movement disorder, tremor, incoordination and dystonia as the results of copper accumulation in the basal ganglia and brain. Besides, patients cannot always fully recover from toxic levels of copper resulting in permanent liver or neurological deficits because of irreversible tissue damage [7,8].

The therapeutic outcome of WD depends upon precise diagnosis and recognition. The presence of various clinical symptoms and difference in age at onset in WD highlight the problem of delaying the time for appropriate treatment leading to severe complications [6,9]. According to previous reports, serum ceruloplasmin measurement is an unreliable diagnostic indicator in patients with liver disease and those who were taking chelation therapy, showing a positive predictive value of only about 5% [10]. In this regard, the generally utilized clinical and laboratory parameters are insufficient to exclude the diagnosis of WD in patients with liver disease of unknown origin. Then we establish a simple and direct biomarker to effectively distinguish WD from other similar diseases, except for the evaluation of ceruloplasmin oxidase activity in the first place.

Fibrinogen is the key coagulation protein and an important determinant of blood viscosity as well as platelet aggregation. In addition, fibrinogen levels were correlated with established risk factors of different chronic diseases including coronary heart disease (CHD), stroke and other vascular problems [11]. There are several mechanisms by which fibrinogen may increase cardiovascular risk. Fibrinogen could bind specifically to activate platelets, leading to platelet aggregation and increased fibrinogen levels may promote fibrin formation. Furthermore, it is also an acute-phase reactant that is increased in inflammatory states [12]. In this regard, measurement of fibrinogen may help in disease prediction or prevention.

Genetic mutational analysis for index cases remains difficult as there are numerous mutations scattered throughout the coding region of the gene within 21 exons [13]. In contrast to the analysis of a selected single parameter, proteomic analysis allows a comprehensive evaluation of hundreds of proteins in parallel [14]. Another reason for focusing on the analysis of protein is that gene sequence does not completely describe the post-translation modification which may be essential for protein function and activity [15]. The proteomic technology comprises a plethora of techniques to resolve two-dimensional electrophoresis (2-DE), quantitate (scanner with software), identify and characterize proteins (sequencing and mass spectrometry), as well as to map information (bioinformatics). In the current study, functional proteome tools were applied to plasma protein analysis related to clinical representations of WD patients.

Since WD patients may present with a variety of clinical characteristics and laboratory alterations, a multidisciplinary approach is necessary. The subjects of WD diagnosed by clinical findings exhibited the obvious decrease in oxidase activity of ceruloplasmin with enzymatic assay. We also presented a 2-DE protein map of plasma from the WD patients and normal controls to develop possible diagnostic markers. According to the results of alignment with protein database, fibrinogen, which may be associated with pathologic processes of liver cirrhosis in WD was characterized and further validated with 2D-immunoblots, immunohistochemistry and analysis of clinical samples. This study aimed at targeting changes in plasma protein profile by proteomic tool to reveal the biological function of plasma protein which may be involved in WD pathology. These proteins might shed light on developing a high throughput screen for potential WD subjects and facilitate diagnosis in this complex disease.

## 2. Results

### 2.1. Enzymatic Analysis of Ceruloplasmin Activity and Evaluation of Protein Carbonylation

**Figure 1:**
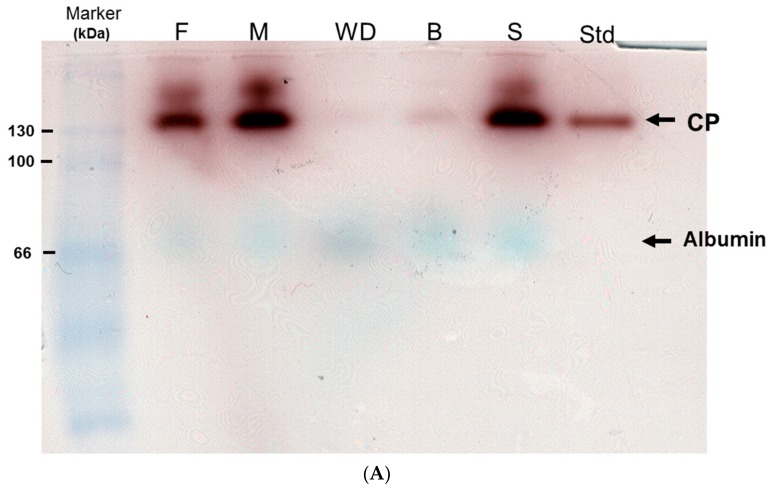

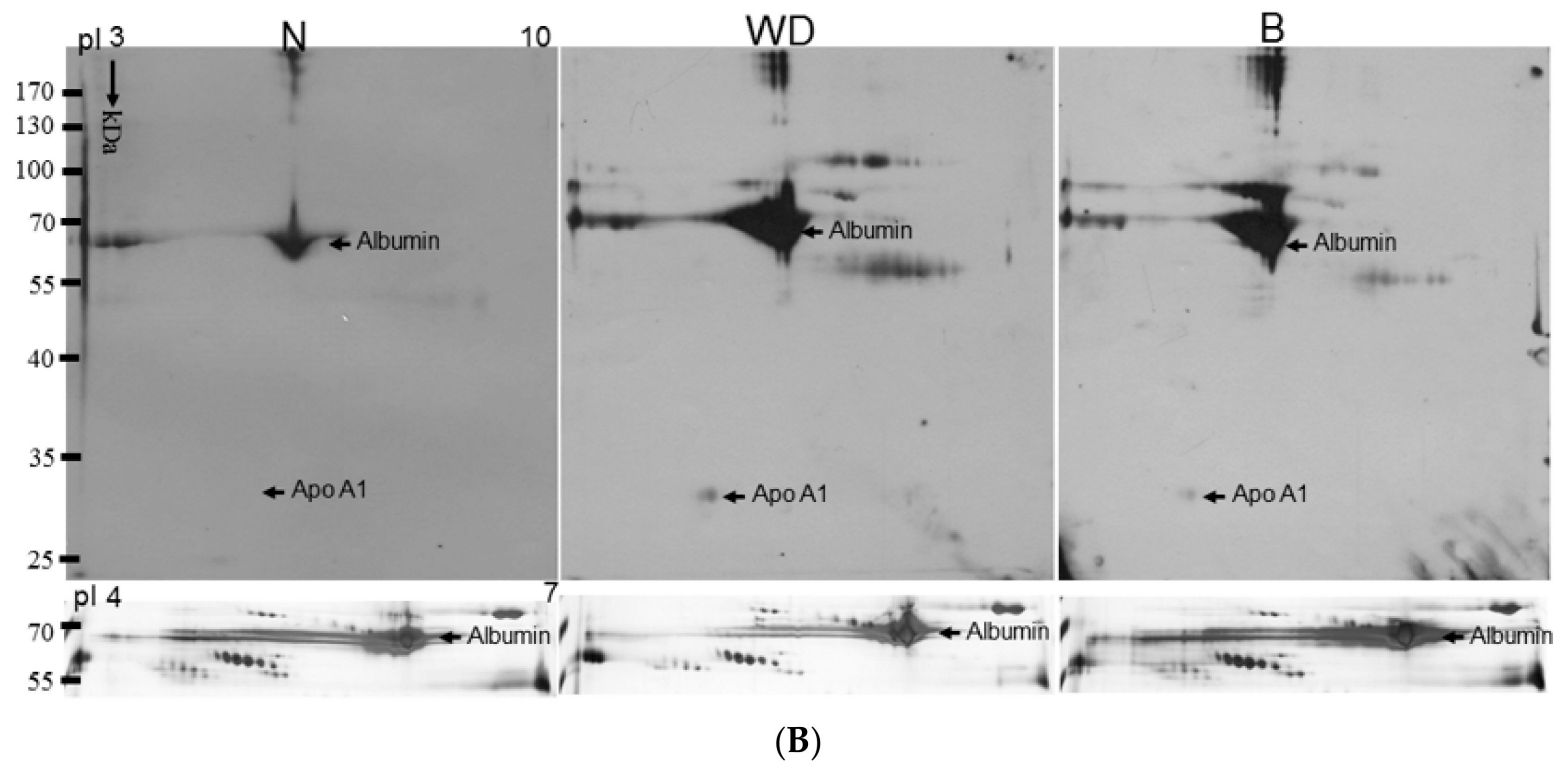
(**A**) In situ oxidase activity of ceruloplasmin oxidase among health donor, heterozygous carriers and homozygous WD patients. The patient (WD) had been undergoing therapy while S and B were diagnosed as health individuals and asymptomatic subjects; however, B started onset six month later. Two paternal samples referred to as F and M, respectively. Marker: protein marker, Std: commercial ceruloplasmin as standard. Albumin is naturally presented as the blue bands after conducting the assay. (**B**) Levels of protein carbonylation. Significantly increased expression of carbonylated proteins were observed in the samples of WD and B compared to the control. Albumin in the lower panels was utilized as a loading control.

Oxidase activity of ceruloplasmin within plasma collected from asymptomatic control (B,S), two parents (F,M) and homozygous WD patient (WD) was assayed simultaneously (Figure 1A). Commercial ceruloplasmin (Std) was used as the standard with 130 kDa in molecular weight. After quantitative analysis divided by internal control (albumin), the enzymatic activity attained to almost 100% in the plasma of sample S; it showed significant decrease by 95% in sample WD and 90% in sample B.

About 20% and 5% reduction of activity were observed in both F and M, respectively, who were considered as heterozygous carriers. Novel WD patient (B) was first identified and introduced to an early regimen because of our findings from this experiment.

Figure 1B shows that protein carbonylation was significantly induced in patients WD and B compared to the control (N) samples. These results implied that high dose of oxidative stress was induced and directly promoted protein oxidation in WD subjects.

### 2.2. Identification of WD-Associated Proteins by Proteomics Analysis

**Figure 2:**
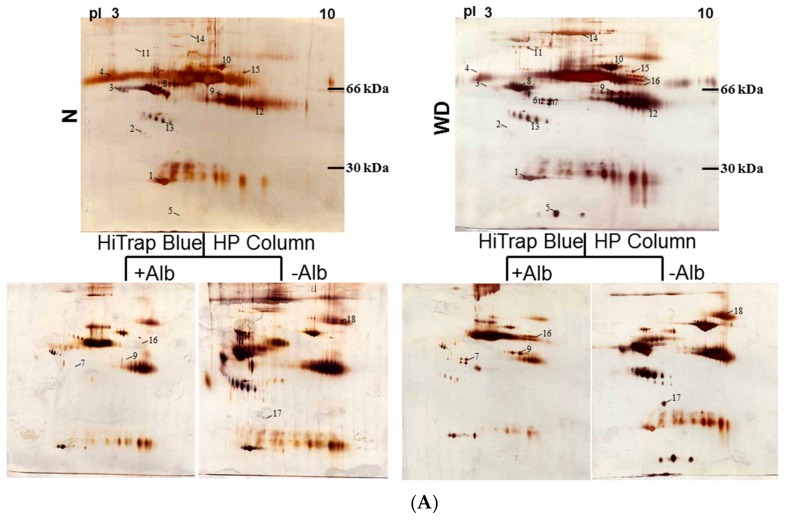

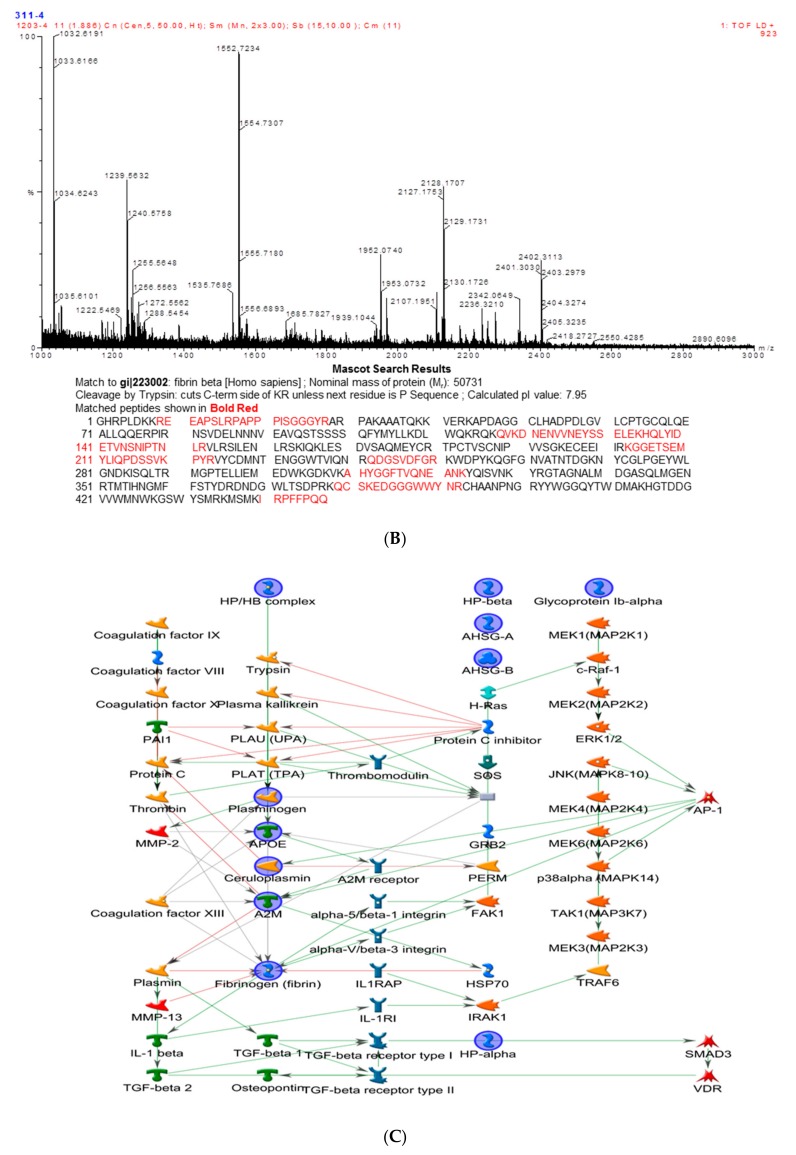

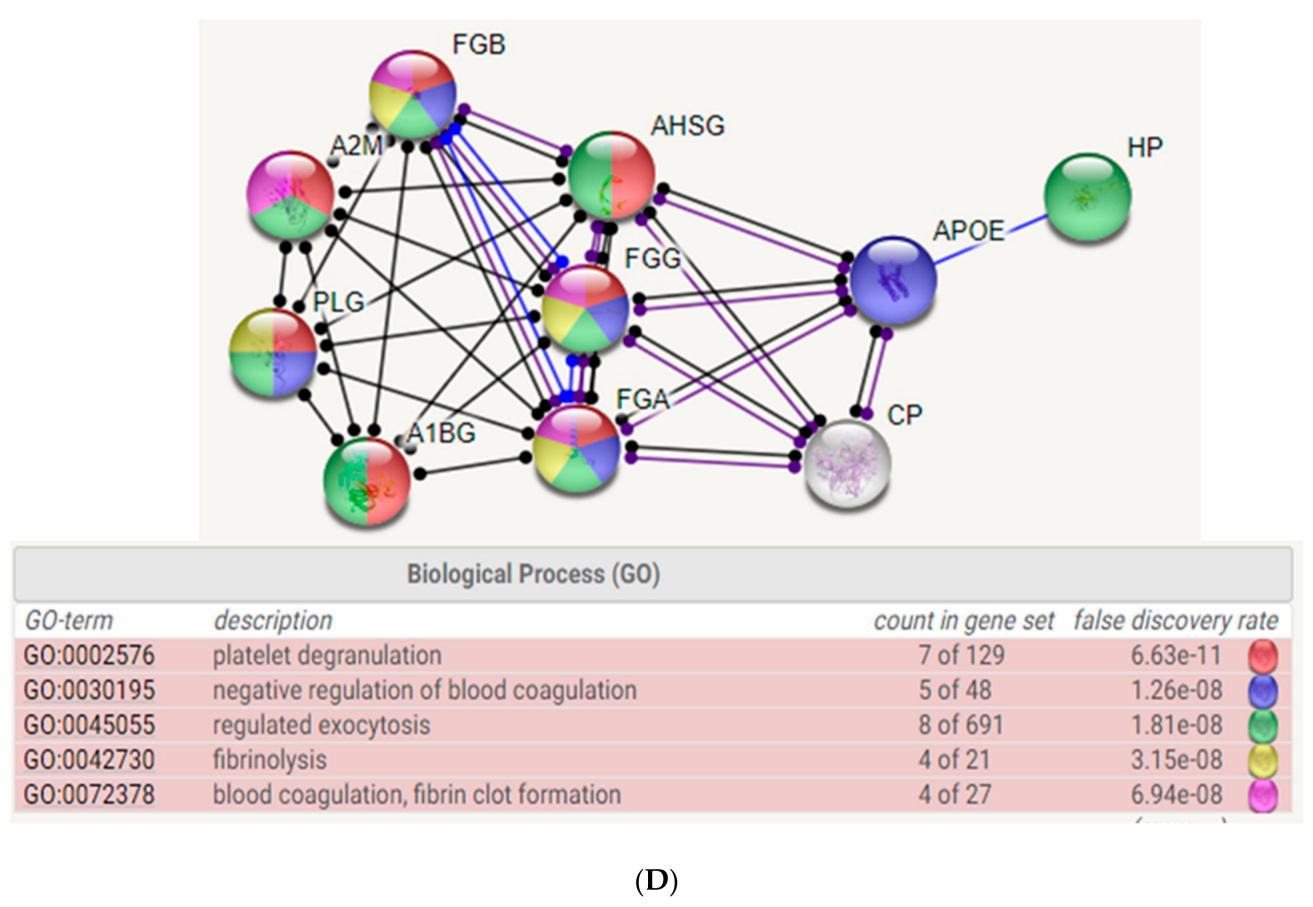
(**A**) Silver-stained 2-DE patterns of plasma and plasma depleted of albumin. N: health donor; WD: Wilson’s disease patients. Most albumins in plasma samples from health donor and WD were removed by using a Millipore Montage Albumin Deplete kit. The protein spots with significantly increasing intensity were labeled as Arabic numerals. Of these thirty one spots, 4 spots which appeared in normal samples were expressed in high level. Conversely, the other 14 spots were found in more abundant quantity for WD samples. (**B**) Peptide mass fingerprints of human fibrinogen β chain by. After 2-DE separation, spots was digested with trypsin and applied to MALDI-TOF mass spectrum which marked 9 in Table 1. By mean of Mascort software, 13 peptides with m/z values could be matched to accession number from Swiss-Prot database. Matched peptides showed with underline within the internal amino acid sequence of fibrinogen β chain. (**C**) Biological network analyses of differentially expressed proteins using MetaCore™ mapping tools. Nodes represent proteins and lines between the nodes indicate direct protein–protein interactions. The various proteins on this map are indicated by different symbols representing the functional class of the proteins. (**D**) Top-ranked pathways from the GeneGo MetaCore pathway analysis. Pathways were ranked according to *p* values.

In order to screen the potential biomarkers associated with WD, 200 μg of plasma from each control and the WD patient were separated by 2-DE with pH value from 3 to 10 and visualized by silver stain (Figure 2 upper panels). Some protein spots were presented clearly after removing the albumin (Figure 2 lower panels).

Using a Nonlinear Progenesis computerized program, a total of 18 protein spots with significant difference between normal controls and WD patients were counted. Of these proteins, four spots appearing in the control samples markedly increased in volume, whereas another 14 spots were found in more abundant quantity for WD samples. As shown in Figure 2A, these protein spots were subsequently identified by peptide mass fingerprinting (PMF) and aligned with the protein sequence in the database as listed in Table 1. Among the identified proteins, two spots (spots 6,7) covering the fibrinogen-gamma family, the protein spots (spots 16) belonging to the fibrinogen-α group and fibrinogen-β chain (spot 9) were also found. Numerous Apo-family protein spots such as Apo A1, Apo J and Apo E, and acute phase proteins which include α2-macroglobulin and haptoglobin α/β chain were explored by this method. At the same time, some proteins considered to relate to the etiology of WD, including antithrombin, complement C3, serotransferrin and other binding proteins were confirmed as well. MS analysis was used to unambiguously identify human fibrinogen as presented in Figure 2B.

### 2.3. Biological Network Analysis

To further elucidate the relationship of the differentially expressed proteins revealed by the 2-DE analysis and their significance in the mechanisms associated with the WD etiology, proteins were analyzed by applying the MetaCore™ software. The algorithm builds biological networks from an uploaded protein list and assigns a biological process to each network which was generated using the shortest-path algorithm to map interactions among proteins identified from the plasma samples. Map Editor was used to build the network based on key proteins which were up- or downregulated in the network. Highlighted lines represent specific, designated pathways. Background lines represent secondary, related biological pathways (Figure 2C). As shown in Figure 2D, protein-protein interaction networks indicated that proteins differentially expressed were primarily related to coagulation system. The following statistically significant networks: platelet degranulation (6.63 × 10^−11^), negative regulation of blood coagulation (1.26 × 10^−8^), regulated exocytosis (1.81 × 10^−8^), fibrinolysis (3.15 × 10^−8^) and blood coagulation (6.94 × 10^−8^). Based on this network, we found that change of fibrinogen caused by WD might be strongly correlated with coagulation system.

### 2.4. Fibrinogen Level Was Detected by 2DE-Immunoblots

**Figure 3:**
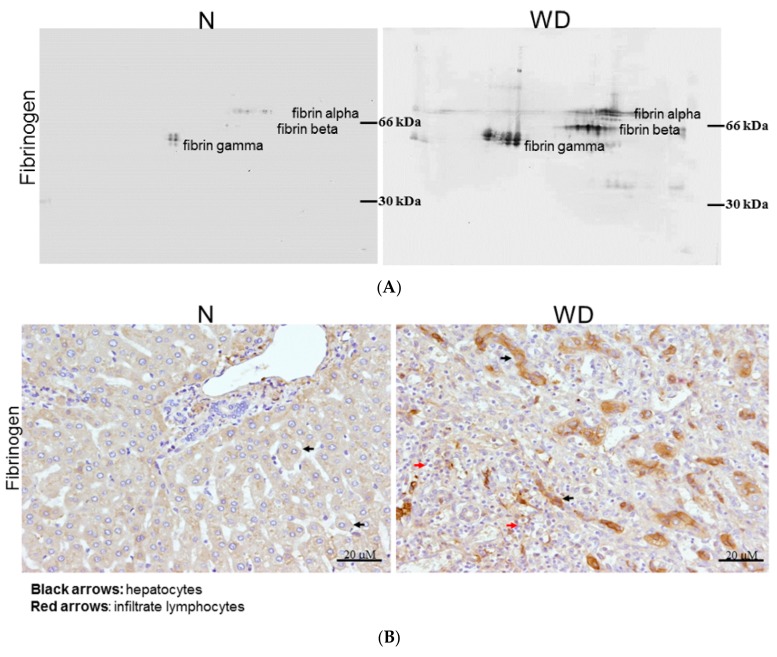

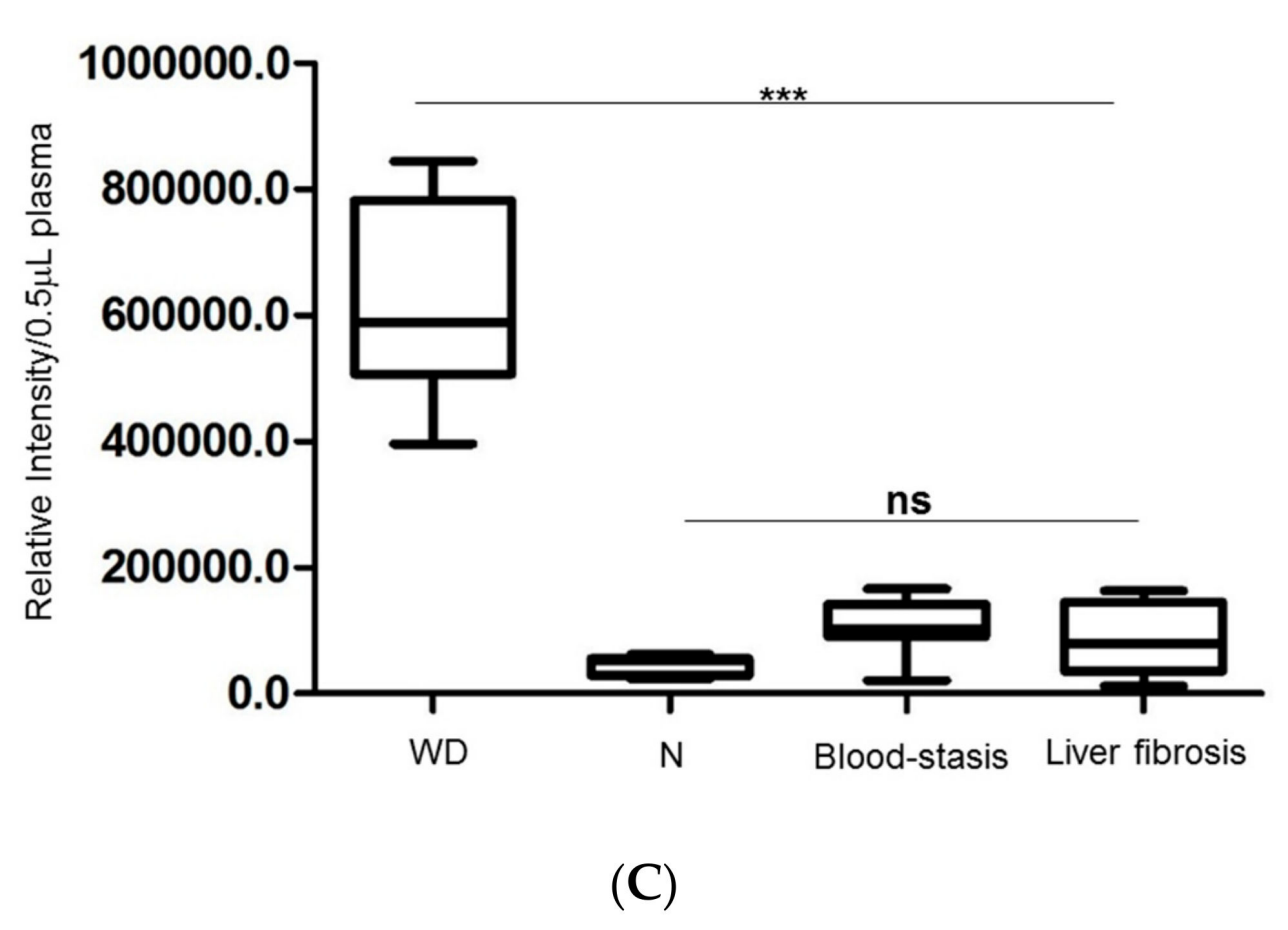
(**A**) Compares the expression of fibrinogen in plasma between normal (N) and WD patient by immunoblots. 2-DE western blot shows the different type of fibrinogen (α, β and γ) with a specific reactive antihuman antibody. (**B**) Immunohistochemical analysis of fibrinogen in the normal part of liver (N) and WD cirrhotic liver are observed under microscope. The hepatocytes were showing obvious staining of fibrinogen in WD patients as black arrows indicate while great amount of inflammatory cells were observed in WD samples as demonstrated by red arrows. However, faint staining of fibrinogen in hepatocytes was found in normal liver sample. (**C**) Changes of fibrinogen level in plasma from clinical subjects with WD, blood-stasis, hepatic fibrosis and healthy donors. Each sample was analyzed in triplicate with specific antibody of fibrinogen in dot blot assay. These results were analyzed statistically by two-way ANOVA and fibrinogen significantly expressed in patients’ plasma with Wilson’s disease (*** *p* < 0.0001).

According to the results from the proteome alternation profiles, we suggest that fibrinogen may be an important index in plasma of WD patients due to hepatic injury, as it was dominant in the Taiwan WD patients. To directly indicate the presence of fibrinogen, we analyzed sera samples from normal controls and WD patients by using 2D-immunoblots. There were seven spots of fibrinogen subunits including fibrinogen α, β, γ chain significantly increased in WD patient while these proteins were expressed in negligible quantity or absent for normal controls (Figure 3A).

### 2.5. Evaluating Fibrinogen Expression in Liver Tissue by Immunohistochemistry

As shown in Figure 3B, fibrinogen distribution in the liver was quite different between normal and WD patients under immunohistochemical assay. We observed that fibrinogen in normal liver was faint staining and localized on the cytoplasm of hepatocytes (Figure 3B; left). Alternatively, the fibrinogen staining became condensed in the perisinusoidal space of the periportal region and cytosol of hepatocytes (Figure 3B; right), which dramatically increased in quantity on WD specimens. The results demonstrated coordinate tendency as in proteomic profiles and western blotting analysis.

### 2.6. Estimation of Fibrinogen Expression in Healthy Controls, Patients with WD and Liver Fibrosis and Blood-Stasis Subjects

Although fibrinogen could be suggested as a potential indicator or biomarker in WD detection, it might also increase in patients with cardiovascular diseases. In order to examine its specific merit, we applied dot-blotting analysis to normal controls, patients with WD and liver fibrosis, and blood-stasis syndrome subjects characterized by a traditional Chinese medical doctor. The levels of fibrinogen were remarkably upregulated (~20-fold) in WD patients compared to healthy donors, patients with liver fibrosis and blood-stasis syndrome (Figure 3C). Based on the above data, we propose that fibrinogen could be feasible in WD diagnosis due to its high specificity.

### 2.7. Characterize the Interplay between Ceruloplasmin and Fibrinogen with Cell Model

**Figure 4:**
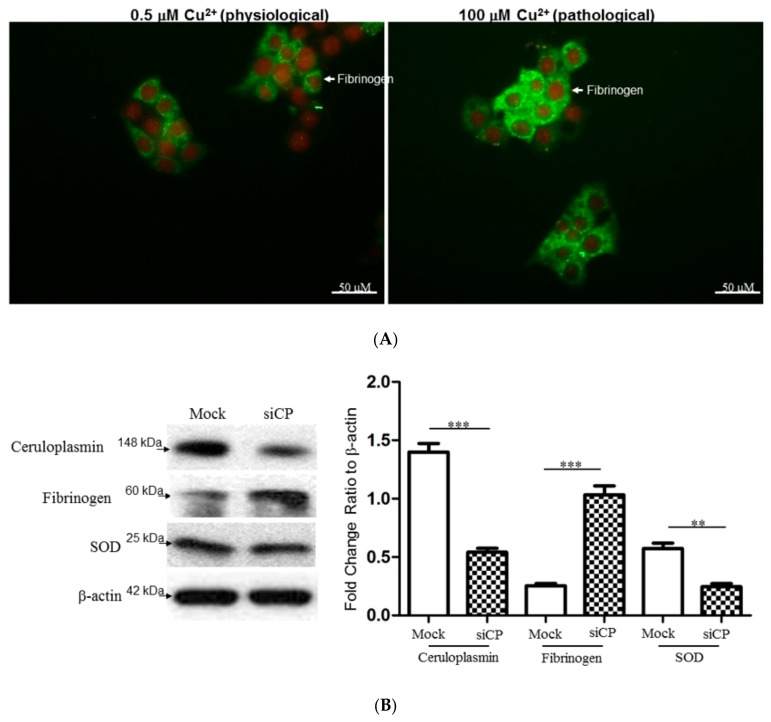

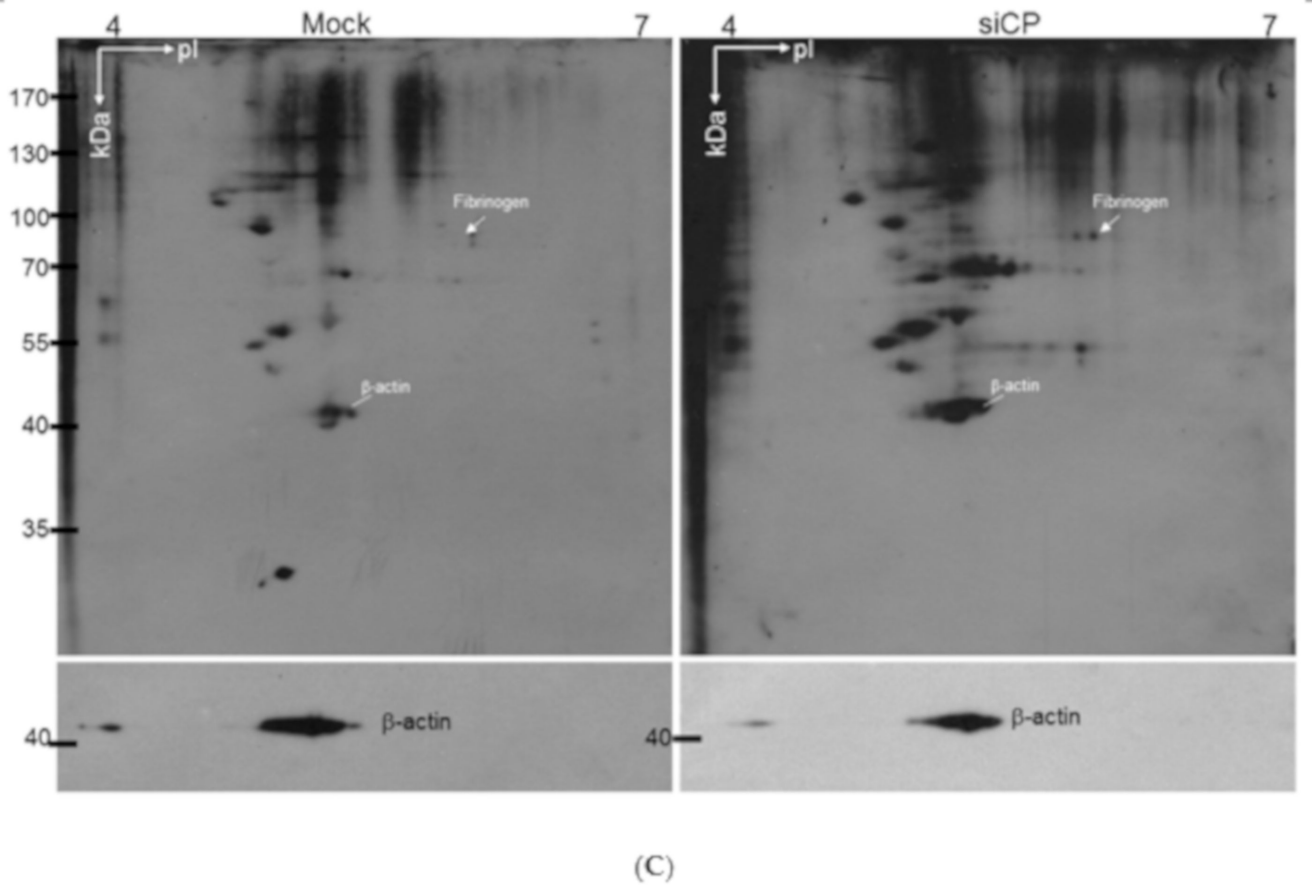
(**A**) Effects of exogenous copper upon changes in the fibrinogen expression of HepG2 cells. After HepG2 cells had been treated with 0.5 and 100 μM copper, the intracellular localizations and level of fibrinogen (green) and nucleus (red) were examined by immunofluorescence microscopy. (**B**) Silence of ceruloplasmin (siCp) treatment induced the protein expression of fibrinogen, while the significant reduction in protein level for SOD was observed under siCp applications. β-actin was used as a loading control. Quantification of the result was presented as the bar diagram and the results represent the mean ± SD of three independent experiments (*** *p* < 0.0001; ** *p* < 0.001). (**C**) Levels of protein carbonylation. Significantly increased expression of carbonylated proteins were observed in the ceruloplasmin-silenced group (siCp) compared to the control (Mock). β-actin performed by Western blot analysis was utilized as the loading control and the individual carbonylated proteins separated by 2-DE analysis then could be normalized to the intensity of the β-actin protein for determining the protein oxidation levels.

To further ascertain the effect of copper and fibrinogen upon hepatic cells, HepG2 cells were exposed to 0.5 and 100 μM copper. As shown in Figure 4A, exogenous administration of high dose of copper markedly increased in fibrinogen level with respect to the sample treated with low dose of copper. Meanwhile, we have utilized ceruloplasmin RNA interference (siCP) to verify the modulation of fibrinogen level and oxidative status in a cell model. Silence of ceruloplasmin significantly enhanced the level of fibrinogen and attenuated antioxidant enzyme protein such as SOD (Figure 4B). Furthermore, increased levels of carbonylated proteins were observed in HepG2 cells transfected with siCP compared to mock-control cells, indicating that the oxidative processes may be initiated under ceruloplasmin suppression (Figure 4C).

## 3. Discussion

The main clinical challenge in the diagnosis of WD is how to characterize this disease earlier and easier by non-invasive method. In current studies, we integrated both ceruloplasmin oxidase activity and proteomics tool, providing evidence for a strategy to screen WD patients effectively whereby only a small amount of plasma is required. Although the serum concentration of ceruloplasmin has been considered as a useful indicator for WD diagnosis, recent studies have demonstrated that immunologic assay of ceruloplasmin may be erroneously estimated because the total amount of ceruloplasmin protein does not reflect ceruloplasmin enzyme activity in the plasma [16,17]. Herein, oxidative function of plasma holoceruloplasmin will be measured via *p*-phenylenediamine oxidation under non-reducing conditions, which is useful for early diagnosis of typically homozygous WD patients and for heterozygous subjects with minor symptoms. Previous experiments on copper depletion in humans suggest that specific enzymatic activity may be a more sensitive indicator of copper accumulation [18,19]. This result supports our hypothesis that measuring enzymatic activity of ceruloplasmin is more accurate and precise than ceruloplasmin concentration assay.

Currently, there are no effective biomarkers or methods available for screening WD, which can lead to neurological disabilities and/or liver cirrhosis. In addition to the measurement of copper accumulation in the liver tissue and ceruloplasmin oxidase activity in plasma, there is still an urgent need to identify other novel biomarkers for improvement in early WD diagnosis [20]. We noted that plasma protein usually reflects physiological changes and is easy to collect. It is reasonable to seek potential diagnostic biomarkers via plasma analysis [21]. In order to comprehensively characterize alterations of the composition in plasma protein occurring to patients with WD, we used 2-DE and MALDI-TOF mass spectrometry to analyze protein profiles herein. From the comparative expression pattern of protein, most meaningful differences of protein between normal controls and WD patients were associated with the hepatic damage.

Fibrinogen, in particular, was found to be abundant in the plasma among all WD patients. It is composed of three structurally different subunits: α, β, and γ chains. In a variety of species, including rodents and human, the γ chain exists in two different forms, called γ-A and γ-B. The γ chain of human fibrinogen has been demonstrated as indicative of tumor-associated fibrin deposition and fibrinolysis [22,23,24]. Interestingly, the relative enrichment with the uploaded data in the bioinformatics experiment indicated that low plasma level of ceruloplasmin has been majorly responsible for the changes in coagulation pathway, resulting in impaired liver function on coagulation [25]. On the other hand, excessive copper accumulating in WD patients destroys the function of fibrinogen and leads to the damage to various organs such as liver and brain. In addition, the network analysis verified that induced fibrinogen is mediated via ROS-ERK1/2/p38/AP-1 signaling pathway. This finding provides an evidence for pro-inflammatory effect of fibrinogen, implying subsequent system injury in WD patients [26]. We also observed significant expression of fibrinogen in liver of WD patients and severe infiltration of lymphocytes through an immunohistochemistry experiment, suggesting the roles of fibrinogen in inducing inflammatory responses (Figure 3B, red arrows). To assess the potent of fibrinogen as a biomarker in early and precise diagnosis of WD, the blood samples obtained from WD patients, liver fibrosis, blood-stasis victims and normal control were applied for evaluation of fibrinogen expression. We found that the plasma content of fibrinogen in subjects with WD is significantly higher than it is in hepatic fibrosis, blood-stasis and normal ones, suggesting fibrinogen may be considered a potential parameter in WD diagnosis.

Biological systems are continuously exposed to reactive oxygen species (ROS) like in the disturbance of copper metabolism in WD subjects [27,28,29]. High doses of ROS determine oxidative stress responsible for serious metabolic dysfunctions and damage to the structures of biological macromolecules such as proteins [30]. Protein carbonylation is considered as a hallmark of oxidative stress [31,32]. The view of 2DE-oxyblots showed that the extent of oxidation in proteins was dramatically upregulated in the case of WD patients compared to the normal control, implying that oxidative stress caused by WD progression would result in increased ROS generation and reduced antioxidant capacity, which caused a visible deterioration in liver and neuron system [33,34,35]. The accumulation of hepatic copper and abrogation of ceruloplasmin synthesis caused by impairment of ATP7B-mediated intracellular copper transport were further simulated by cellular experiments. Our results indicated that high dose copper would induce overproduction of fibrinogen and ceruloplasmin diminution mediated by siRNAs significantly promoted oxidative stress by downregulating the SOD level [36]. These findings highlight the unique properties of fibrinogen in cellular targets and signal transduction pathways related to WD development.

Up to now, no single diagnostic test can exclude or confirm WD with 100% certainty. The prognosis for survival in the majority of WD patients is favorable if they could be provided accurate therapy before complications resulting from copper deficiency. In this paper, we combined two methods: ceruloplasmin oxidase activity and fibrinogen level for achieving an early and rapid diagnosis in WD for the first time.

## 4. Conclusions

**Figure 5:**
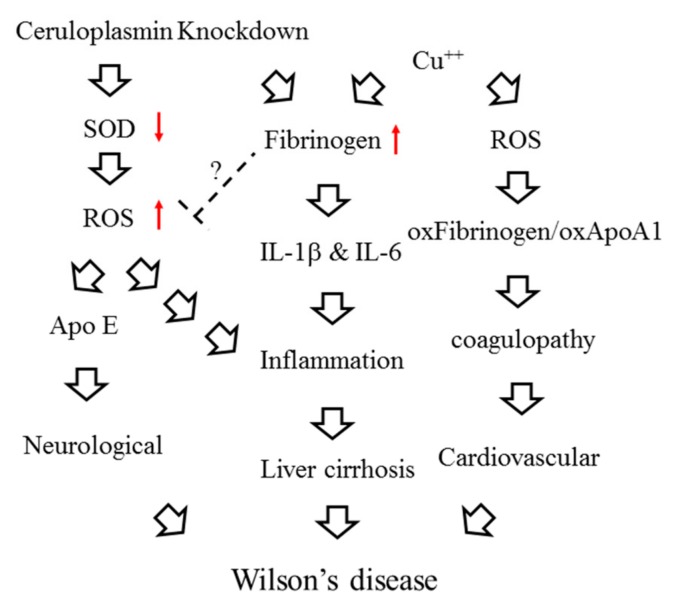
Schematic diagram of the interactions between ceruloplasmin, fibrinogen and oxidative stress. The red arrow up means increase and the red arrow down indicates decrease.

In summary, decreased expression and impaired function of ceruloplasmin in WD patients could bring about copper accumulation and subsequent oxidative stress, which consecutively induces expression of fibrinogen and elicits inflammatory responses as well as dysregulation of coagulation cascades (Figure 5).

## 5. Materials and Methods

### 5.1. Subjects

All clinical data of WD patients were provided by Wen-Neng Chang. The Committee on Research Involving Human Subjects of the Chang Gung Memorial Hospital in Kaohsiung, Taiwan, approved this study. Informed consent was obtained from all subjects in this study. The clinical samples belong to the same family which is including of two parents (F,M), brother and sister (B,S) and homozygous WD patient (WD). Of note, subject B without WD symptoms starts onset six month later after conducting experiment while F, M and S are asymptomatic. Plasma from patients and healthy volunteers was collected, aliquots and storage at −80 °C until assayed.

### 5.2. In Situ Oxidase Activity of Ceruloplasmin

Plasma (5 μL) was mixed with Laemmli sample buffer lacking DTE, and was separated without prior heating by SDS-PAGE. The gel was then equilibrated with 50 vol. of 0.05% Triton X-100 in 10% (*wt*/*vol*) glycerol, soaked for an equal time in 5 vol. of 3 mM *p*-phenylenediamine dihydrochloride/100 mM sodium acetate, pH 5.7, 1 mM NaN_3_, and air-dried in the dark at room temperature for 24 h [37]. Albumin present in the plasma samples will be presented as a blue color under the above treatment.

### 5.3. Two-Dimensional Polyacrylamide Gel Electrophoresis (2-DE)

Approximately 200 μg of plasma were solubilized in the rehydration buffer containing 8 M urea, 2% CHAPS, 0.002% bromophenol blue, 2% IPG buffer pH 3–10 and 65 mM DTE. The samples were then separated by the Immobiline Drystrip on the IPGphor III System (GE Healthcare, Göteborg, Sweden) for the first dimension. The 2-DE was carried out on 10% acrylamide gels at 30 mA/gel [38]. All gels were visualized by silver staining and then scanned using an Imagescanner (GE Healthcare, Göteborg, Sweden). All experiments were repeated three times to confirm the reproducibility.

### 5.4. Albumin Removal

The protocol was followed as described in the Montage Albumin Deplete Kit user guide (Merck Millipore, Burlington, MA, USA). Flow-through contained the albumin-depleted plasma fraction. Albumin was recovered from the column with 2-DE rehydrated buffer. Both fractions were further analyzed by 2-DE platform.

### 5.5. Tryptic In-Gel Digestion of Proteins & MALDI-TOF Mass Spectrometry

Protein spots of interest were excised and in-gel digested with trypsin as previously described [39]. Briefly, tryptic peptides were acidified with 0.5% TFA and loaded onto an MTP AnchorChip™ 600/384 TF (Bruker-Daltonik, Bremen, Germany). The MS analysis was performed on an Ultraflex™ MALDI-TOF mass spectrometer (Bruker-Daltonik, Bremen, Germany) and the monoisotopic peptide masses were applied for database searches with the MASCOT search engine [http://www.matrixscience.com].

### 5.6. Biological Network Analysis Using Metacore™

Applied MetaCore™ software (vers. 5.2 build 17389, GeneGo, St. Joseph, MI, USA) was utilized to explore ontological classes and associated pathways which were denoted for the protein targets characterized by the mass fingerprint [39].

### 5.7. D-Oxyblot and 2D-Immunoblots

Following IEF, IPG strips were incubated in 2N HCl with 10 mM DNPH at 25 °C for 20 min. Next, samples were washed with 2M Tris-Base/30% glycerol for 15 min and the protein was separated according to molecular weight as described above. The 2-DE gels were then transferred to a PVDF membrane which was incubated overnight at 4 °C with the primary antibody for DNPH (Molecular Probes, Eugene, Oregon, USA) or fibrinogen (DAKO, Santa Clara, CA, USA). The blots were washed and incubated with goat anti-rabbit IgG conjugated HRP for 2 h. Enhanced chemiluminescence (Millipore, Burlington, MA, USA) was applied for detection [40].

### 5.8. Immunohistochemistry

Paraffin-embedded tissue blocks were sectioned in 2-μm slices and placed on slides coated with poly-l-lysine. IHC with a fibrinogen (1:400-diluted in phosphate-buffered saline) was applied to sections as previously described [39]. Thereafter, sections were counter-stained with Mayer’s hematoxylin for 2 min, and slides were mounted with mounting medium and evaluated under a light microscope (BX51, Olympus, Tokyo, Japan). Digital photomicrographs were then processed with DP-72 (Olympus).

### 5.9. Analysis of Plasma Fibrinogen in Clinical Samples

Blood samples from 10 healthy volunteers, patients with WD, blood-stasis syndrome and hepatic fibrosis were collected. 0.5 µL of plasma was spotted onto pieces of nitrocellulose paper (Millipore, Burlington, MA, USA) and allowed to dry under room temperature. Paper was then incubated in anti-fibrinogen antibody for 1 h at RT. After three washes with TBST, anti-rabbit IgG conjugated AP was applied to react further for 1 h at RT. After washing as before and rinse with ddH_2_O, paper was developed using NBT/BCIP for 5 min [41]. The target dots were scanner, analyzed and quantified using GeneTools software (version 4.03, Syngene, Cambridge, UK). All experiments were repeated more than twice.

### 5.10. Immunofluorescence

HepG2 cells were treated with copper (0.5 and 100 μM) for 24 h, and were then fixed in ice-cold methanol for 10 min at 4 °C. After washing in PBS, cells were permeabilized with 0.1% Triton-X100 in PBS for 10 min at room temperature. Cells were then incubated with primary antibodies against fibrinogen and rinsed three times in PBS. Cells were subsequently exposed to a Fluorescein-5-Isothiocyanate conjugated secondary antibody (Santa Cruz Biotechnology, Dallas, TX, USA). After incubation, cells were rinsed in PBS three times and nuclei were counterstained with propidium iodide (Sigma-Aldrich, St. Louis, MO, USA) for 1 min. After washing three times, the cells were maintained with mounting medium and observed by Olympus IX71 fluorescence microscope (Olympus, Tokyo, Japan) under a DP72 PhotoImage system (Olympus, Tokyo, Japan) [39].

### 5.11. siRNA Administration

6 × 10^5^ cells/dish HepG2 cells were cultured with antibiotic-free medium for 6 h and transfected with a mixture containing either 0.5 μg/well scrambled siRNA (mock) or a mixture of three ceruloplasmin siRNAs (smart pool; Invitrogen, Waltham, MA, USA), Opti-MEM, 8 μL/well Lipofectamine 2000 (Invitrogen). The cells were harvested and subjected to following experiments after 48 h post-transfection.

### 5.12. Western Blot Analysis

The protein obtained from the various experiments was isolated using 1 × cell lysis buffer (Cell Signaling, Danvers, MA, USA) and the concentration was determined with the Bradford Protein Assay Kit (AMRESCO, Solon, OH, USA). Protein lysates were then investigated by Western blot analyses as previously described [39,42]. The specific antibodies used in the current study were listed as follows: CP (abcam, Cambridge, UK), Fibrinogen, SOD and β-actin. The band intensity was quantified by using GeneTools software and the level of β-actin was performed as internal control.

### 5.13. Statistical Analysis

All values were presented as the mean ± SD. Statistical analysis of the mean values was carried out with the ANOVA test using the SPSS software (version 12.0, SPSS, Chicago, IL, USA). Differences were considered significant at *p* < 0.001. Data was confirmed through three repetitions.

## Figures and Tables

**Table 1:** List of identified protein spots.

Spot No.	Protein Name	Accession number	Score (Coverage)	Mw (kDa)/pI	Function
1	Apo A1	P02647	215 (61%)	30.76/5.56	Participates in the reverse transport of cholesterol from tissues to the liver for excretion by promoting cholesterol efflux from tissues and by acting as a cofactor for the lecithin cholesterol acyltransferase (LCAT).
2	Apo J (clusterin)	P10909	38 (19%)	49.34/6.27	Prevents stress-induced aggregation of blood plasma proteins.
3	α2-HS-glycoprotein	P02765	72 (28%)	40.10/5.43	Promotes endocytosis, possesses opsonic properties and influences the mineral phase of bone. Shows affinity for calcium and barium ions.
4	α1-B-glycoprotein	P04219	130 (43%)	54.81/5.65	.
5	Haptoglobin α chain	P00738	80 (51%)	13.74/6.10	Haptoglobin also acts as an antioxidant, has antibacterial activity, and plays a role in modulating many aspects of the acute phase response.
6	Fibrinogen-γ-A chain	P02679	158 (58%)	50.82/5.70	Together with fibrinogen-α (FGA) and fibrinogen-β (FGB), polymerizes to form an insoluble fibrin matrix.
7	Fibrinogen-γ-B chain	P02679	156 (56%)	52.11/5.37	Together with fibrinogen-α (FGA) and fibrinogen-β (FGB), polymerizes to form an insoluble fibrin matrix.
8	antithrombin	P01008	182 (58%)	53.04/6.32	Most important serine protease inhibitor in plasma that regulates the blood coagulation cascade. AT-III inhibits thrombin, matriptase-3/TMPRSS7, as well as factors IXa, Xa and XIa.
9	Fibrinogen-	P02675	225 (62%)	56.58/8.54	Cleaved by the protease thrombin to yield monomers which, together with fibrinogen-α (FGA) and fibrinogen-γ (FGG), polymerize to form an insoluble fibrin matrix. Fibrin deposition is also associated with infection, where it protects against IFNG-mediated hemorrhage.
10	Serotransferrin	P02787	93 (37%)	74.28/6.63	Transferrins are iron binding transport proteins which can bind two Fe^3+^ ions in association with the binding of an anion, usually bicarbonate.
11	α1-antiproteinase	P01009	148 (47%)	46.88/5.37	Inhibitor of serine proteases. Its primary target is elastase, but it also has a moderate affinity for plasmin and thrombin.
12	Immunoglobulin α2 heavy chain	P01876	133 (37%)	54.82/6.02	Constant region of immunoglobulin heavy chains. Immunoglobulins, also known as antibodies, are membrane-bound or secreted glycoproteins produced by B lymphocytes.
13	Haptoglobin β chain	P00738	126 (46%)	38.87/6.26	Haptoglobin captures, and combines with free plasma hemoglobin to allow hepatic recycling of heme iron and to prevent kidney damage.
14	α2-microglobulin	P01023	172 (25%)	164.6/6.00	Is able to inhibit all four classes of proteinases by a unique ‘trapping’ mechanism.
15	Complement C3	P01024	107 (24%)	188.57/6.02	C3 plays a central role in the activation of the complement system.
16	Fibrinogen-α	P02671	127 (38%)	94.97/5.70	Cleaved by the protease thrombin to yield monomers which, together with fibrinogen-β (FGB) and fibrinogen-γ (FGG), polymerize to form an insoluble fibrin matrix.
17	ApoE	P02649	82 (43%)	36.15/5.65	APOE is an apolipoprotein, a protein associating with lipid particles, that mainly functions in lipoprotein-mediated lipid transport between organs via the plasma and interstitial fluids
18	Plasminogen	P00747	72 (23%)	90.57/7.04	Plasmin dissolves the fibrin of blood clots and acts as a proteolytic factor in a variety of other processes including embryonic development, tissue remodeling, tumor invasion, and inflammation.

Database: NCBIprot 20180429 (152462470 sequences; 55858910152 residues).

## Abbreviations

WD	Wilson’s disease
DNPH	2,4-Dinitrophenylhydrazine
MALDI-TOF-MS	Matrix-Assisted Laser Desorption/ Ionization Time of Flight Mass Spectrometry
SOD	Superoxide dismutase
2-DE	Two-dimensional electrophoresis
CP	Ceruloplasmin
PMF	Peptide mass fingerprinting
ROS	Reactive oxygen species
IPG	Immobilized pH gradient
IHC	Immunohistochemistry
NBT	p-Nitrobluetetrazolium chloride
BCIP	5-Bromo-4-chloro-3-indolyl phosphate
DTE	Dithioerythritol
SDS-PAGE	Sodium dodecyl sulfate-polyacrylamide gel electrophoresis.

## References

[1] Dzieżyc K., Litwin T., Chabik G., Gramza K., Członkowska A. (2014). Families with Wilson’s disease in subsequent generations: Clinical and genetic analysis. Mov. Disord..

[2] Medici V., Weiss K.H. (2017). Genetic and environmental modifiers of Wilson disease. Handb. Clin. Neurol..

[3] Kluska A., Kulecka M., Litwin T., Dziezyc K., Balabas A., Piatkowska M., Paziewska A., Dabrowska M., Mikula M., Kaminska D. (2019). Whole-exome sequencing identifies novel pathogenic variants across the ATP7B gene and some modifiers of Wilson’s disease phenotype. Liver Int..

[4] Bhattacharjee A., Chakraborty K., Shukla A. (2017). Cellular copper homeostasis: Current concepts on its interplay with glutathione homeostasis and its implication in physiology and human diseases. Metallomics.

[5] Bandmann O., Weiss K.H., Kaler S.G. (2015). Wilson’s disease and other neurological copper disorders. Lancet Neurol..

[6] Roberts E.A. (2018). Update on the Diagnosis and Management of Wilson Disease. Curr. Gastroenterol. Rep..

[7] Wu F., Wang J., Pu C., Qiao L., Jiang C. (2015). Wilson’s disease: A comprehensive review of the molecular mechanisms. Int. J. Mol. Sci..

[8] Hordyjewska A., Popiołek Ł., Kocot J. (2014). The many “faces” of copper in medicine and treatment. Biometals.

[9] Lorincz M.T. (2012). Recognition and treatment of neurologic Wilson’s disease. Semin. Neurol..

[10] Macintyre G., Gutfreund K.S., Martin W.R., Camicioli R., Cox D.W. (2004). Value of an enzymatic assay for the determination of serum ceruloplasmin. J. Lab. Clin. Med..

[11] Danesh J., Lewington S., Thompson S.G., Lowe G.D., Collins R., Kostis J.B., Wilson A.C., Folsom A.R., Wu K., Benderly M. (2005). Plasma fibrinogen level and the risk of major cardiovascular diseases and nonvascular mortality: An individual participant meta-analysis. JAMA.

[12] Stec J.J., Silbershatz H., Tofler G.H., Matheney T.H., Sutherland P., Lipinska I., Massaro J.M., Wilson P.F., Muller J.E., D’Agostino R.B. (2000). Association of fibrinogen with cardiovascular risk factors and cardiovascular disease in the Framingham Offspring Population. Circulation.

[13] Ferenci P. (2006). Regional distribution of mutations of the ATP7B gene in patients with Wilson disease: Impact on genetic testing. Hum. Genet..

[14] Greco V., Piras C., Pieroni L., Urbani A. (2017). Direct Assessment of Plasma/Serum Sample Quality for Proteomics Biomarker Investigation. Methods Mol. Biol..

[15] Gallart-Palau X., Serra A., Sze S.K. (2015). Uncovering Neurodegenerative Protein Modifications via Proteomic Profiling. Int. Rev. Neurobiol..

[16] Cauza E., Maier-Dobersberger T., Polli C., Kaserer K., Kramer L., Ferenci P. (1997). Screening for Wilson’s disease in patients with liver diseases by serum ceruloplasmin. J. Hepatol..

[17] Xu R., Jiang Y.F., Zhang Y.H., Yang X. (2018). The optimal threshold of serum ceruloplasmin in the diagnosis of Wilson’s disease: A large hospital-based study. PLoS ONE.

[18] Vassiliev V.B., Kachurin A.M., Beltramini M., Rocco G.P., Salvato B., Gaitskhoki V.S. (1997). Copper depletion/repletion of human ceruloplasmin is followed by the changes in its spectral features and functional properties. J. Inorg. Biochem..

[19] Merle U., Eisenbach C., Weiss K.H., Tuma S., Stremmel W. (2009). Serum ceruloplasmin oxidase activity is a sensitive and highly specific diagnostic marker for Wilson’s disease. J. Hepatol..

[20] Hahn S.H. (2014). Population screening for Wilson’s disease. Ann. NY Acad. Sci..

[21] Geyer P.E., Kulak N.A., Pichler G., Holdt L.M., Teupser D., Mann M. (2016). Plasma Proteome Profiling to Assess Human Health and Disease. Cell Syst..

[22] Litvinov R.I., Weisel J.W. (2016). What Is the Biological and Clinical Relevance of Fibrin?. Semin Thromb. Hemost..

[23] Lin Y., Liu Z., Qiu Y., Zhang J., Wu H., Liang R., Chen G., Qin G., Li Y., Zou D. (2018). Clinical significance of plasma D-dimer and fibrinogen in digestive cancer: A systematic review and meta-analysis. Eur. J. Surg. Oncol..

[24] Pieters M., Wolberg A.S. (2019). Fibrinogen and fibrin: An illustrated review. Res. Pract. Thromb. Haemost..

[25] Schaefer M., Weber L., Gotthardt D., Seessle J., Stremmel W., Pfeiffenberger J., Weiss K.H. (2015). Coagulation Parameters in Wilson Disease. J. Gastrointest. Liver Dis..

[26] Davalos D., Akassoglou K. (2012). Fibrinogen as a key regulator of inflammation in disease. Semin Immunopathol..

[27] Zischka H., Lichtmannegger J. (2014). Pathological mitochondrial copper overload in livers of Wilson’s disease patients and related animal models. Ann. NY Acad. Sci..

[28] Clayton P.T. (2017). Inherited disorders of transition metal metabolism: An update. J. Inherit. Metab. Dis..

[29] Yu Y., Guerrero C.R., Liu S., Amato N.J., Sharma Y., Gupta S., Wang Y. (2016). Comprehensive Assessment of Oxidatively Induced Modifications of DNA in a Rat Model of Human Wilson’s Disease. Mol. Cell Proteom..

[30] Rosenfeld M.A., Vasilyeva A.D., Yurina L.V., Bychkova A.V. (2018). Oxidation of proteins: Is it a programmed process?. Free Radic. Res..

[31] Fedorova M., Bollineni R.C., Hoffmann R. (2014). Protein carbonylation as a major hallmark of oxidative damage: Update of analytical strategies. Mass. Spectrom. Rev..

[32] Baraibar M.A., Friguet B. (2013). Oxidative proteome modifications target specific cellular pathways during oxidative stress, cellular senescence and aging. Exp. Gerontol..

[33] Scheiber I.F., Brůha R., Dušek P. (2017). Pathogenesis of Wilson disease. Handb. Clin. Neurol..

[34] Scheiber I.F., Mercer J.F., Dringen R. (2014). Metabolism and functions of copper in brain. Prog. Neurobiol..

[35] Boukhenouna S., Wilson M.A., Bahmed K., Kosmider B. (2018). Reactive Oxygen Species in Chronic Obstructive Pulmonary Disease. Oxid. Med. Cell Longev..

[36] Fukai T., Ushio-Fukai M. (2011). Superoxide dismutases: Role in redox signaling, vascular function, and diseases. Antioxid. Redox Signal..

[37] Levin L.A. (2002). Ceruloplasmin detection by SDS-PAGE, immunoblotting, and in situ oxidase activity. Methods Mol. Biol..

[38] Wang P.W., Chang W.N., Lu C.H., Chao D., Schrag C., Pan T.L. (2006). New insights into the pathological mechanisms of cerebrotendinous xanthomatosis in the Taiwanese using genomic and proteomic tools. Proteomics.

[39] Pan T.L., Wang P.W., Huang C.C., Yeh C.T., Hu T.H., Yu J.S. (2012). Network analysis and proteomic identification of vimentin as a key regulator associated with invasion and metastasis in human hepatocellular carcinoma cells. J. Proteomics..

[40] Wang P.W., Wu T.H., Pan T.L., Chen M.H., Goto S., Chen C.L. (2018). Integrated Proteome and Cytokine Profiles Reveal Ceruloplasmin Eliciting Liver Allograft Tolerance via Antioxidant Cascades. Front. Immunol..

[41] Pan T.L., Goto S., Lin Y.C., Lord R., Chiang K.C., Lai C.Y., Chen Y.S., Eng H.L., Cheng Y.F., Tatsuma T. (1999). The fas and fas ligand pathways in liver allograft tolerance. Clin. Exp. Immunol..

[42] Pan T.L., Wang P.W., Leu Y.L., Wu T.H., Wu T.S. (2012). Inhibitory effects of Scutellaria baicalensis extract on hepatic stellate cells through inducing G2/M cell cycle arrest and activating ERK-dependent apoptosis via Bax and caspase pathway. J. Ethnopharmacol..

